# A clavicular overuse injury in a teenage male gymnast: a rare case report

**DOI:** 10.3389/fped.2026.1734056

**Published:** 2026-02-04

**Authors:** Tiago Guedes Almeida, Giacomo De Marco, Oscar Vazquez, Christina Steiger, Romain Dayer, Sana Boudabbous, Dimitri Ceroni

**Affiliations:** 1 Pediatric Orthopedics Unit, Pediatric Surgery Service, Geneva University Hospitals, Geneva, Switzerland; 2 Radiology Department, Geneva University Hospitals, Geneva, Switzerland

**Keywords:** clavicle, orthopaedics, overuse injury, paediatrics, physeal injury, sports, sternoclavicular joint

## Abstract

Overuse injuries are frequent in young athletes, particularly in gymnasts. However, physeal injuries of the medial clavicle remain exceptionally rare. We report an unusual case highlighting the diagnostic value of magnetic resonance imaging (MRI). A 12.5-year-old male gymnast presented with progressive pain and swelling of the right sternoclavicular joint. Clinical evaluation was complemented by radiographs, MRI, and CT scans to assess the lesion. Imaging revealed irregularity and widening of the medial clavicular physis, associated with synovial capsular dehiscence and enlargement of the articular facet, without signs of infection or inflammation. CT confirmed disturbed endochondral ossification, consistent with a repetitive physeal injury. This case illustrates the susceptibility of growth plates to repetitive stress in paediatric athletes. Medial clavicular physeal injury should be considered in gymnasts with chronic sternoclavicular pain. MRI plays a key role in early and accurate diagnosis.

## Introduction

The number of children and adolescents participating in sports activities has increased dramatically in recent decades. Indeed, participating in organised sports is now regarded as a rite of passage in childhood development. Growing participation in organised competitive sports has inevitably led to a surge in acute and overuse injuries (1–4). Overuse injuries typically result from repetitive mechanical stress, particularly when recovery time is insufficient. They are common among teenagers and adolescents because these young athletes are experiencing rapid physical growth and an imbalance between muscle strength and flexibility.

Gymnastics is a popular discipline in this age group, often practised at a competitive level. During gymnastics practice, skills and routines are repeated again and again, placing extraordinary stress on their upper limbs' growing physes. Thus, high-level child gymnasts' arms are subject to tremendous torsional forces with axial loading. The most common stress injuries among these children are widely recognised as stress fractures, traction apophysitis, repetitive physeal injuries and epiphyseal osteochondrosis. Indeed, the two latter injuries are of particular concern, given their potential to disrupt growth. We describe a 12.5-year-old male gymnast patient with a rare repetitive physeal injury of the clavicle's medial extremity, resulting in a functional disorder and pain in the sternoclavicular joint. This case report contributes valuable insights into the clinical presentation and diagnosis of this problem in paediatric patients.

## Case report

**Figure 1:**
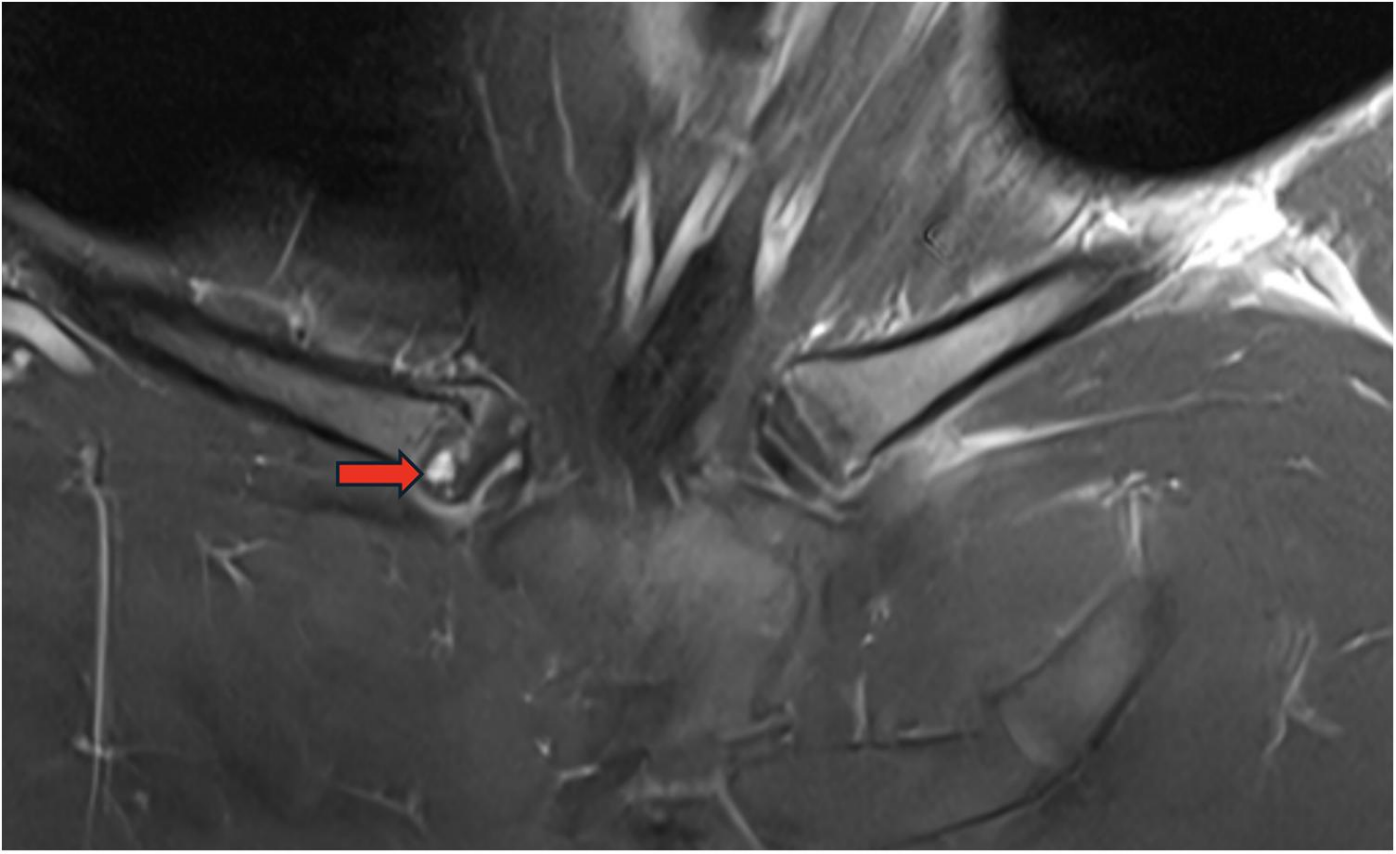
T1-weighted spin echo coronal sequence with frequency-selective fat saturation demonstrated a dysplastic appearance of the right clavicle's proximal epiphysis with a heterogeneous signal (red arrow).

**Figure 2:**
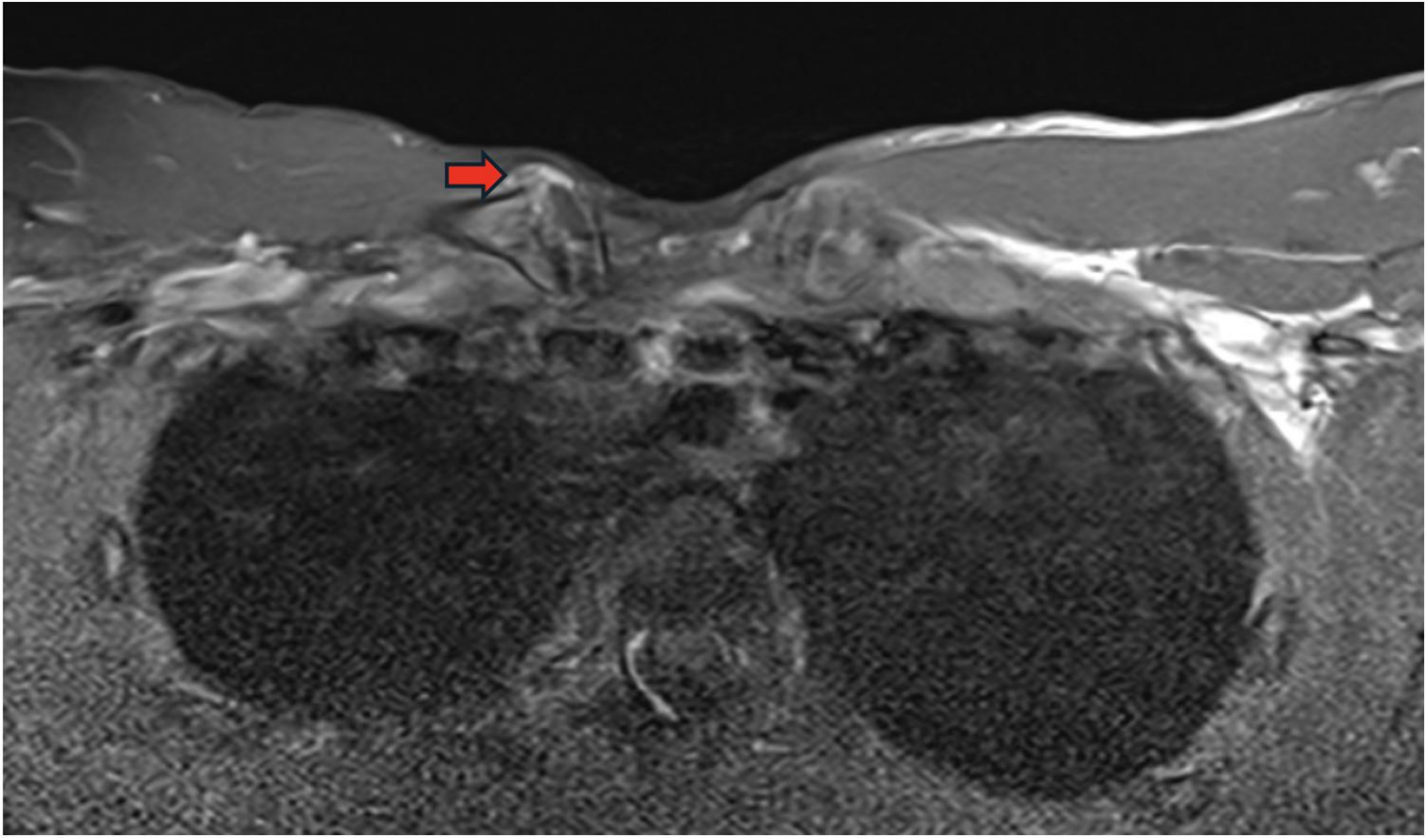
T1-weighted spin echo axial sequence with frequency-selective fat saturation showed an irregularity with widening of the medial physis of the clavicle. There was also an anterior bulging of the sternoclavicular joint capsule suggesting a slight intra-articular effusion (red arrow).

**Figure 3:**
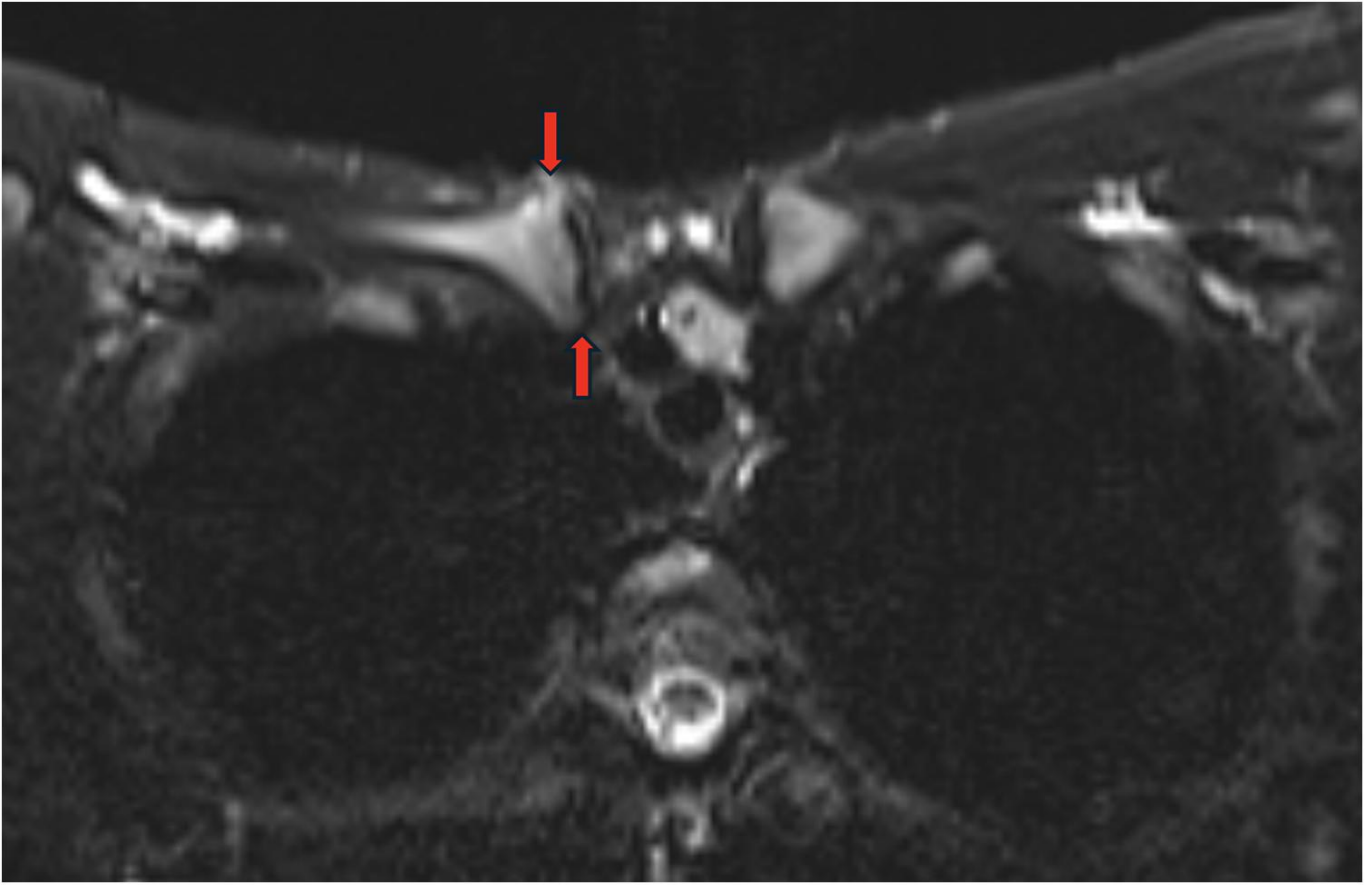
MRI showing a widening of the articular facet without erosion or signs of bone oedema (red arrows).

**Figure 4:**
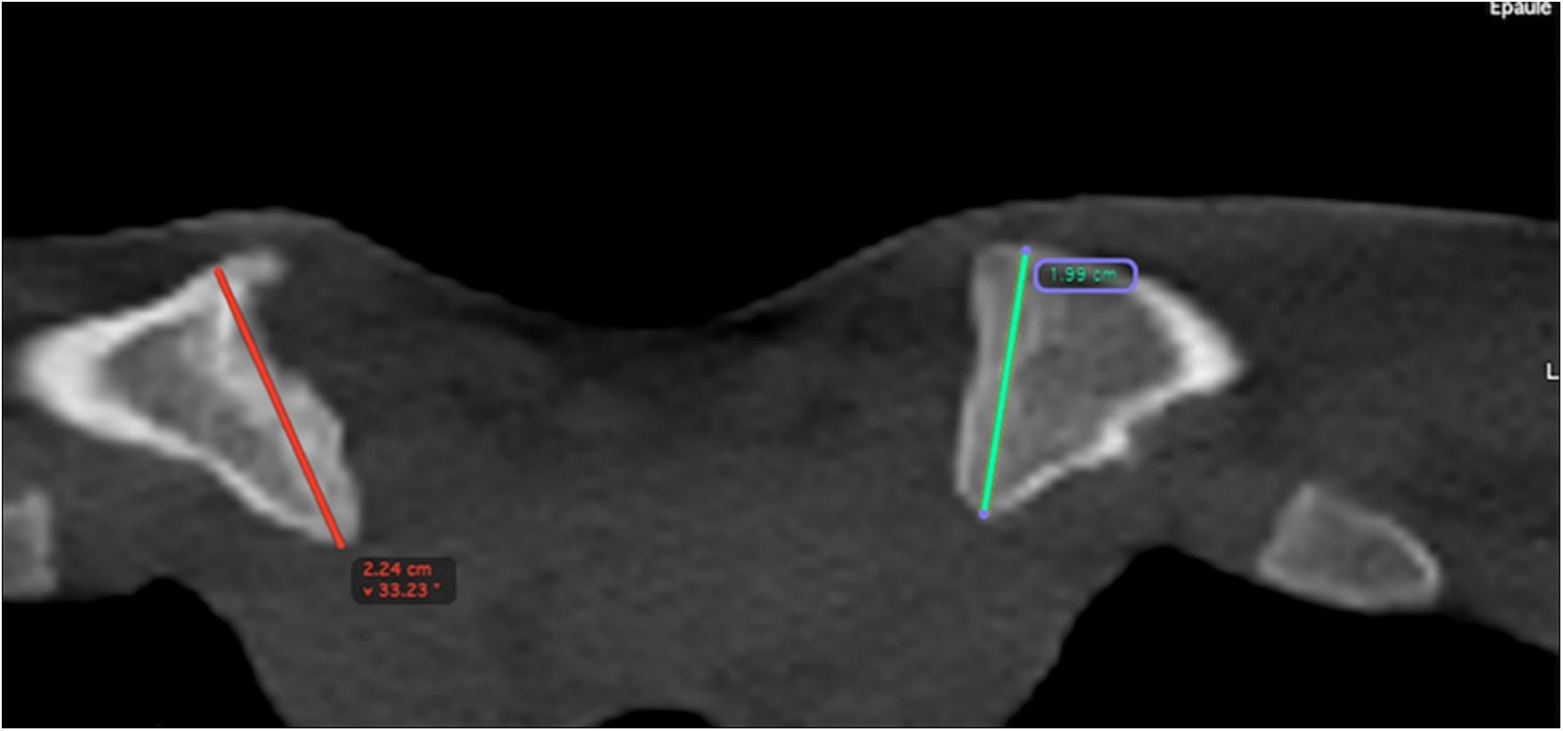
CT showing the irregularity of the metaphysis with enlarged aspect (red line).

A healthy 12.5-year-old boy was referred to our university hospital paediatric orthopaedics unit by a specialist sports medicine paediatrician because of a painful swelling in his right sternoclavicular joint. In his anamnesis, the patient reported no trauma, no history suggestive of inflammatory arthropathy in his family and no previous infectious arthritis. However, the patient reported participating in high-level artistic gymnastics, with a training schedule of 15–20 h per week. His complaints revolved around the chronic and progressive pain he felt during gymnastics activities at the level of the right sternoclavicular joint. Locally, the patient had also noticed the development of a protrusion on the clavicle's medial extremity. Pain was triggered by direct palpation of the sternoclavicular joint and by movements of this articulation. Conventional radiography revealed an enlargement of the right clavicle's medial metaphysis and the irregular appearance of the physis. Magnetic resonance imaging was performed with images acquired at 1.5 T (Avanto, Siemens, Erlangen, Germany) using the following sequences: T1-weighted turbo spin-echo (coronal); T1 short tau inversion recovery (STIR) axial; T2-weighted turbo spin-echo (sagittal); T2 STIR (coronal and axial); and post-contrast injection [10 mL of gadoteric acid (Dotarem, Guerbet, France)] T1-weighted spin echo with frequency-selective fat saturation (coronal) and T1-weighted turbo spin-echo (coronal and axial). T1-weighted spin echo coronal sequence with frequency-selective fat saturation demonstrated a dysplastic appearance of the right clavicle's proximal epiphysis with a heterogeneous signal (Figure 1). T1-weighted spin echo axial sequence with frequency-selective fat saturation showed an irregularity with widening of the medial physis of the clavicle. There was also an anterior bulging of the sternoclavicular joint capsule suggesting a slight intra-articular effusion (Figure 2). Finally, a widening of the articular facet with no erosion or signs of bone oedema was present on T2 axial turbo spin-echo STIR sequence (Figure 3). However, there was no inflammation *per se* within the joint cavity. A computed tomography scan confirmed the irregularity of the metaphysis, which appeared enlarged (Figure 4). Many differential diagnoses had to be excluded given this atypical clinical presentation. A traumatic physeal injury was quickly ruled out due to the absence of reported trauma and the absence of fracture on the MRI. The infectious hypothesis (whether osteomyelitis or septic arthritis) could also be ruled out based on clinical, biological, and radiological findings. The absence of a primary joint involvement seemed unlikely to be compatible with an inflammatory joint involvement, and the radiological appearance of the lesion was not suggestive of a SAPHO spectrum disease. Finally, the radiological imaging was not suspicious for neoplastic or tumor-like lesions. This examination mainly highlighted an endochondral ossification disorder originating in the physis, which appeared scalloped. Thus, the diagnosis made was that of a repetitive physeal injury of the medial clavicle extremity. The patient was treated conservatively (NSAIDs), and put to rest, avoiding physical activities involving the upper limbs for 3 months. This led to partial pain relief**,** which spontaneously recurred when artistic gymnastics training resumed. After 12 months of this ineffective treatment, the patient decided to redirect his sporting activities and turn to football. Since then, the pain has almost completely disappeared, and the patient has resumed another sporting activity which he considers satisfactory.

## Discussion

Although overuse is well recognised as a factor leading to chronic strain and inflammation of the sternoclavicular joint in adults, there is little or no information about the equivalent mechanism among skeletally immature athletes (1, 3, 5–11). To the best of our knowledge, the case described here is the first to report a repetitive overuse lesion presenting as a physeal injury of the clavicle's medial extremity. These sites are only considered to be 20%–50% as strong as other structures (5). Thus, it is not the sternoclavicular joint that constitutes the limb's weak link, as in adults, but the physis itself.

Although inactivity causes difficulties for proper bone growth in paediatric populations, so does joint overuse (12–15). In skeletally immature athletes, repetitive loading preferentially affects growth cartilage, which represents the primary biomechanical weak point. These injuries are generally correlated with inadequate preparation, poor training, a rising intensity of training and incomplete physical maturation. They are often observed among poorly conditioned athletes during periods of rapid increases in the tempo of training and among elite athletes training at consistently high tempos. Another factor is that children are often not accorded enough time to rest during and between practice sessions. It has been demonstrated that sports activities requiring high-level training involving repetitive loading can disrupt endochondral ossification of the long bones and alter metaphyseal perfusion. This is not an insignificant phenomenon as it can interfere with mineralisation's processing of hypertrophied chondrocytes in the provisional calcification zone, resulting in a widening of the primary physis and the deposit of “tongue-like” focal extensions of non-mineralised cartilage in the metaphysis and its subsequent deformation. Injuries similar to those observed in skeletally immature children participating in high-level sports and sustaining repetitive and axial load traumas have been produced by disrupting the metaphyseal blood flow in experimental animal models (16–19). Indeed, the case reported here perfectly matches descriptions of lesions called repetitive physeal injuries. However, it is important to remember that we must distinguish these from epiphyseal osteochondrosis, which constitutes an overload problem that can also be encountered among gymnasts, particularly at the level of the capitulum (20).

Artistic gymnastics is a breathtaking sport to watch, but it subjects the upper limbs to tremendous torsional forces and axial loading due to repetitive weight bearing. Overuse injuries in gymnasts are more common among females than males, and the most affected joints are wrists and elbows (3, 5–7, 9–11). The skills practised during gymnastics put extraordinary stresses on the growing ends of the radius and ulna, on carpal bones and bones in the hand, and on the many ligaments that stabilise these structures. The most common stress injuries include stress fractures, repetitive physeal injuries and epiphyseal osteochondrosis, all of which risk disrupting growth. Common gymnastics injuries of the upper limbs include shoulder instability, ulnar collateral ligament injuries, capitellar osteochondritis dissecans and several wrist pathologies (4). It is crucial, therefore, that these athletes progress under medical supervision as part of overarching strategies designed to prevent musculoskeletal pain and injuries.

## Conclusion

Gymnasts are exposed to a high risk of acute and overuse injuries, but also of developing musculoskeletal pain. The most common stress injuries among skeletally immature gymnasts are stress fractures, repetitive physeal injuries and epiphyseal osteochondrosis. The most frequent gymnastics injuries or lesions of the upper limbs involve the wrist, the elbow and the shoulder. The case described here involved a repetitive overload of the sternoclavicular joint that led to an atypical form of repetitive physeal injury.

## Data Availability

The original contributions presented in the study are included in the article/Supplementary Material, further inquiries can be directed to the corresponding author.

## References

[1] Alaia EF Rosenberg ZS Rossi I Zember J Roedl JB Pinkney L Growth plate injury at the base of the coracoid: MRI features. Skeletal Radiol. (2017) 46(11):1507–12. 10.1007/s00256-017-2736-0 28756567

[2] Bedoya MA Jaramillo D Iwasaka-Neder J Laor T. Stressed or fractured: MRI differentiating indicators of physeal injury. Skeletal Radiol. (2024) 53(11):2437–47. 10.1007/s00256-024-04670-y 38557698

[3] Caine D DiFiori J Maffulli N. Physeal injuries in children’s and youth sports: reasons for concern? Br J Sports Med. (2006) 40(9):749–60. 10.1136/bjsm.2005.017822 16807307 PMC2564388

[4] Connolly LP Jaramillo D. Imaging of sports injuries in children and adolescents. Radiol Clin North Am. (2001) 39(4):773–90. 10.1016/S0033-8389(05)70310-9 11549170

[5] Delgado J Jaramillo D Chauvin NA. Imaging the injured pediatric athlete: upper extremity. RadioGraphics. (2016) 36(6):1672–87. 10.1148/rg.2016160036 27726752

[6] Kocher MS Waters PM Micheli LJ. Upper extremity injuries in the paediatric athlete. Sports Med. (2000) 30(2):117–35. 10.2165/00007256-200030020-00005 10966151

[7] Ogawa K Inokuchi W Matsumura N. Physeal injuries of the coracoid process are closely associated with sports activities: a systematic review. Orthop J Sports Med. (2020) 8(12):2325967120967914. 10.1177/2325967120967914 33403213 PMC7747117

[8] Ojeda PI Kresse ME Lazo CR Delucia TA Gaskin CM. Proximal fibular physeal stress injury: a known entity in an unusual location. Pediatr Radiol. (2023) 53(1):175–8. 10.1007/s00247-022-05444-1 35867111

[9] Roy S Caine D Singer KM. Stress changes of the distal radial epiphysis in young gymnasts: a report of twenty-one cases and a review of the literature. Am J Sports Med. (1985) 13(5):301–8. 10.1177/036354658501300503 4051086

[10] Shih C Chang CY Penn IW Tiu CM Chang T Wu JJ. Chronically stressed wrists in adolescent gymnasts: mR imaging appearance. Radiology. (1995) 195(3):855–9. 10.1148/radiology.195.3.7754021 7754021

[11] Yong-Hing K Wedge JH Bowen CV. Chronic injury to the distal ulnar and radial growth plates in an adolescent gymnast. A case report. J Bone Joint Surg Am. (1988) 70(7):1087–9. PMID: 3403578. 3403578

[12] DiFiori JP. Overuse injury of the physis: a “growing” problem. Clin J Sport Med. (2010) 20(5):336–7. 10.1097/JSM.0b013e3181ebb55d 20818188

[13] Ecklund K Jaramillo D. Imaging of growth disturbance in children. Radiol Clin North Am. (2001) 39(4):823–41. 10.1016/S0033-8389(05)70313-4 11549173

[14] Malina RM. Critical review: exercise as an influence upon growth: review and critique of current concepts. Clin Pediatr (Phila). (1969) 8(1):16–26. 10.1177/000992286900800106 4883071

[15] Nguyen JC Markhardt BK Merrow AC Dwek JR. Imaging of pediatric growth plate disturbances. RadioGraphics. (2017) 37(6):1791–812. 10.1148/rg.2017170029 29019753

[16] Jaramillo D Laor T Zaleske DJ. Indirect trauma to the growth plate: results of MR imaging after epiphyseal and metaphyseal injury in rabbits. Radiology. (1993) 187(1):171–8. 10.1148/radiology.187.1.8451408 8451408

[17] Laor T Hartman AL Jaramillo D. Local physeal widening on MR imaging: an incidental finding suggesting prior metaphyseal insult. Pediatr Radiol. (1997) 27(8):654–62. 10.1007/s002470050206 9252430

[18] Maynard JA Pedrini-Mille A Pedrini VA Vailas AC. Morphological and biochemical effects of strenuous exercise on immature long bones. Iowa Orthop J. (1995) 15:162–7. PMID: 7634027. 7634027 PMC2329060

[19] Trueta J Amato VP. The vascular contribution to osteogenesis: iII. Changes in the growth cartilage caused by experimentally induced ischaemia. The Journal of Bone and Joint Surgery British Volume. (1960) 42-B(3):571–87. 10.1302/0301-620X.42B2.367 17533673

[20] Dwek JR Chung CB. A systematic method for evaluation of pediatric sports injuries of the elbow. Pediatr Radiol. (2013) 43(S1):120–8. 10.1007/s00247-012-2585-x 23478927

